# Fluid-Attenuated Inversion Recovery Vascular Hyperintensities in Transient Ischemic Attack within the Anterior Circulation

**DOI:** 10.1155/2020/7056056

**Published:** 2020-02-18

**Authors:** Bei Ding, Yong Chen, Hong Jiang, Huan Zhang, Juan Huang, Hua-wei Ling

**Affiliations:** ^1^Department of Radiology, Ruijin Hospital, School of Medicine, Shanghai Jiao Tong University, 197, Ruijin Er Road, 200025 Shanghai, China; ^2^Department of Neurosurgery, Ruijin Hospital, School of Medicine, Shanghai Jiao Tong University, 197, Ruijin Er Road, 200025 Shanghai, China

## Abstract

**Purpose:**

The aim of the present study was to evaluate the relationship of fluid-attenuated inversion recovery (FLAIR) vascular hyperintensities (FVH) with haemodynamic abnormality and severity of arterial stenosis in patients with transient ischemic attack (TIA) of the carotid artery system. *Patients and Methods*. Consecutive inpatients (*N* = 38) diagnosed with TIAs of the carotid system in a 4-year period (2014–2017) were retrospectively analysed in our study and divided into FVH-negative and FVH-positive groups based on the presence of FVH sign. Each inpatient had undergone magnetic resonance imaging (MRI) followed by computed tomography (CT) perfusion imaging studies. We investigated the degree of arterial stenosis, number of stenosis, watershed regions, and related CT perfusion indexes, including hypoperfusion regions, mean transit time (MTT), cerebral blood flow (CBF), and cerebral blood volume (CBV). Spearman rank correlation was performed between FVHs score, the degree of arterial stenosis, and CT perfusion indexes with significant difference.

**Results:**

Thirty-one patients (81.6%) observed with FVH sign were assigned to the FVH-positive group. The hypoperfusion regions, MTT, and CBF values were significantly different between the FVH-negative group and FVH-positive groups. Spearman correlation analysis showed significant positive correlations between hypoperfusion regions, MTT, and FVHs scores (*r* = 0.755 and 0.674, respectively, *p* < 0.01); a moderate negative correlation was found between CBF and FVHs scores (*r* = 0.755 and 0.674, respectively, *p* < 0.01); a moderate negative correlation was found between CBF and FVHs scores (*r* = 0.755 and 0.674, respectively, *p* < 0.01); a moderate negative correlation was found between CBF and FVHs scores (

**Conclusion:**

Hyperintense vessels on FLAIR were closely associated with hypoperfused regions, MTT, and CBF values, which indicated that the presence of FVHs could be an important and convenient imaging marker of haemodynamic impairment in patients with TIA.

## 1. Introduction

Vascular hyperintensities on fluid-attenuated inversion recovery (FLAIR) images were first described in a series of patients with acute stroke and subacute stroke in 1999 [1] and termed “FLAIR vascular hyperintensity” (FVH). FVHs are described as focal, tubular, or serpentine hyperintensities often seen in the subarachnoid space against the relative hypointensity of cerebrospinal fluid (CSF). This neuroimaging sign has been observed both in large vessel steno-occlusive disease due to atherosclerosis and in other diseases, such as transient ischemic attack (TIA) and Moyamoya disease [2–5]. The mechanism underlying the hyperintense vessels on FLAIR imaging remains to be established. Possible explanations suggested for their appearance include stationary blood and slow antegrade or retrograde filling of the leptomeningeal collateral circulation [6–9]. However, in the past decade, most studies have concentrated on the appearance of FVH in patients with acute cerebral infarction, and most authors agree that FVH is associated with major vessel occlusion or severe stenosis, as well as impaired haemodynamics. Few reports have been concerned with the significance of FVH in patients with TIA. The relationship between FHVs, the degree of arterial stenosis, and haemodynamic abnormality has not been assessed. Given the potential importance of FHV, the purpose of this study is to clarify in TIA patients which factor plays a critical role in FHV by using computed tomography (CT) perfusion imaging and magnetic resonance angiography (MRA).

## 2. Materials and Methods

### 2.1. Selection Criteria

We performed a retrospective analysis of consecutive inpatients admitted with hemispheric TIAs in our institution from June 2014 to April 2017. TIA was defined as a sudden, focal neurological deficit of less than 24 hours in duration without acute infarction on imaging. Imaging data were selected from all patients who had (1) transient neurologic symptoms judged by clinical neurologists at the end of the evaluation to have a possible vascular aetiology, (2) arterial stenosis of the carotid artery system that was confirmed by MRA, and (3) MR scans performed within 48 hours of symptom onset. Exclusion criteria were as follows: (1) Patients receiving any thrombolytic agent or an investigational drug therapy prior to magnetic resonance imaging (MRI) scanning. (2) Patients with recent acute stroke. (3) Patients who had severe coexisting or terminal systemic disease.

### 2.2. Image Acquisition

MRI scans were acquired using 1.5TGE Signa Horizon scanners equipped with enhanced gradient systems (GE Medical Systems, Waukesha, WI). FLAIR: 256 × 256 matrices, TI = 2500 ms, TR = 8500 ms, TE = 140 ms, field of view = 240 mm, slice thickness/gap = 5.0/0.0 mm. Diffusion weighted imaging (DWI): spin echo-echo planar imaging (SE-EPI), TR = 4800 ms, TE = 82 ms, 256 × 256 matrices, field of view = 240 mm, slice thickness/gap = 5.0/0.0 mm. Three-dimensional time-of-flight MRA of the intracranial vessels and a 3D contrast-enhanced MRA of internal carotid artery (ICA) circulation with intravenous administration of gadolinium-diethylenetriamine penta-acetic acid (Gd-DTPA) were performed.

CT perfusion images were obtained using a multirow detector CT scanner (GE Discovery CT750 HD, GE Medical Systems). Perfusion images were acquired with intravenous injection of 50 ml nonionic iodinated contrast medium containing 370 mg iodine per millilitre at an injection rate of 4–5 ml/s through a 20-gauge intravenous line using an automatic injector. At 5–7 s after initiation of the injection, a cine (continuous) scan was initiated with the following parameters: 80 kV, 200 mA, 512 × 512 matrix, field of view 25 cm, slice thickness 5 mm, 0.5 s per rotation for a duration of 45 to 50 s.

### 2.3. Data Processing and Scoring System

For post-processing, all CT perfusion data were transferred to an Advantage Windows Workstation (version 4.6; General Electric Medical Systems) equipped with a perfusion software package (CT Perfusion 4; GE Medical Systems). The perfusion maps of the cerebral blood volume (CBV), cerebral blood flow (CBF), and mean transit time (MTT) were generated off-line at the workstation.

**Figure 1:**
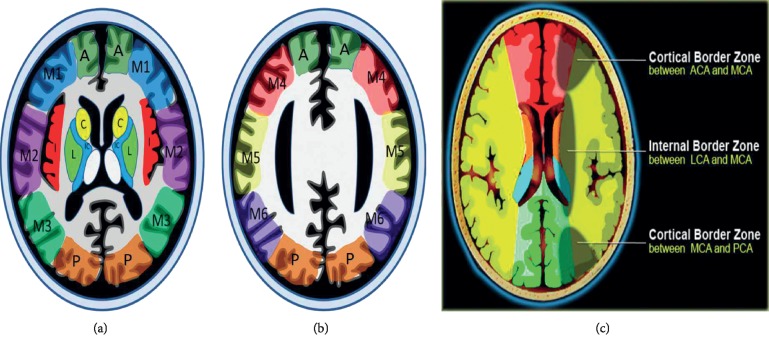
The Alberta Stroke Programme Early CT Score (ASPECTS) regions at the ganglionic (a) and supraganglionic (b) levels. The regions are the six cortical MCA regions (M1–M6), the insular cortex (I), the lentiform nuclei (L), the internal capsule (IC), the caudate head (C), ACA region (A), PCA region (P), and the three types of border zones (c).

Two experienced neuroradiologists who were blinded to clinical information reviewed FLAIR images to score FHVs and CT perfusion images to reveal perfusion deficit regions, which were defined as brain tissue with MTT > 6 s in consensus. The hypoperfusion scores ranged from zero to 15 points according to the corresponding arterial territories. As shown in Figure 1, the Alberta Stroke Programme Early Computerized Tomography Score (ASPECTS) is commonly used to assess CT scans of patients with acute stroke, but we modified its territorial definitions for patients with TIAs on MTT maps; in addition, we added the anterior cerebral arterial region, posterior cerebral arterial region, and the three types of border zones. For each patient, a rounded region of interest (ROI) was manually drawn by the two radiologists in a selected area where most obviously prolonged MTT was detected. ROIs were placed carefully to avoid arterial and venous structures. These measurements were repeated twice. The results were averaged, and the corresponding values of CBV and CBF were also measured.

Hyperintense vessels on FLAIR imaging were defined as linear or serpentine-appearing hyperintensity relative to grey matter in the subarachnoid space that corresponded with a typical arterial course. FVH score was calculated as described in a study from Olindo et al. [3]. All axial T2-FLAIR images were analysed. Slices with no FVH were scored as 0, and those with one or more FVH were scored as 1. As 16 images were analysed, the resulting FVH score was 0–16. Watershed regions were defined as number of hypoperfusion areas presenting at cerebral watershed, including anterior cortical type, posterior cortical type and subcortical type.

According to the standard of the North American Symptomatic Carotid Endarterectomy Trial (NASCET) [10], the degree of cerebral arterial stenosis was classified as a 4-grade scale: grade 1 (mild, 0–29%), grade 2 (moderate, 30–69%), grade 3 (severe, 70–99%), and grade 4 (occlusion, 100%). Arterial stenosis was graded by comparing the diameter of the maximally stenosed artery with the diameter of the more proximal normal segment of the same vessel.

### 2.4. Statistical Analysis

All analyses were performed with the Statistical Package for the Social Sciences, Version 19.0 software (SPSS, Chicago, Illinois). We used Student's *t*-test for continuous variables and contingency tables with the Fisher exact test for qualitative variables. The Kappa statistic was used for assessing the extent of agreement of interobserver reproducibility for stenosis grade. We applied Spearman rank correlation coefficient to assess the correlations between FHVs, perfusion parameters with significant difference, hypoperfusion regions, and stenosis grade. A 2-tailed value of *p* < 0.05 was considered to be statistically significant.

## 3. Results

**Figure 2:**
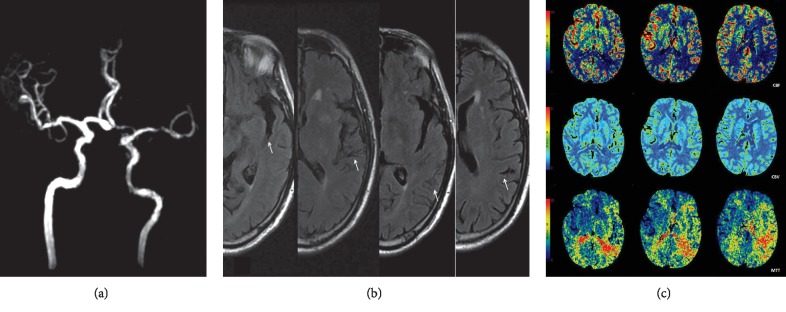
Fluid-attenuated inversion recovery vascular hyperintensity in a patient with left middle cerebral artery severe stenosis (M1). (a) MRA showed severe stenosis of the left MCA M1. (b) Dot-like and serpentine hyperintense signal of the middle cerebral artery branches in the left Sylvian fissure and the sulci of the left fronto-parietal lobes (arrows). (c) CT perfusion imaging showed severe hypoperfusion with prolonged MTT and decreased CBF and CBV values in bilateral cortical border zones and the left side of MCA territories.

**Figure 3:**
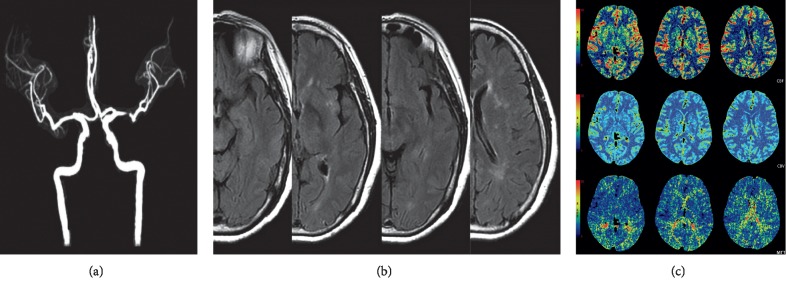
FVH absent in a patient with left middle cerebral artery severe stenosis (M1). (a) MRA showed a severe stenosis of left MCA M1. (b) No hyperintense signal of the middle cerebral artery branch was found in the corresponding fissure or the sulci of the left fronto-parietal lobes. (c) CT perfusion imaging showed hypoperfusion with prolonged MTT in the bilateral anterior and posterior cortical border zones, and no clearly decreased CBF or CBV values were found.

Ninety-seven patients with TIA were retrospectively collected and 59 were excluded (25 patients receiving thrombolytic agents or an investigational drug therapy prior to MRI scanning; 20 patients with recent acute stroke; 14 patients who had severe coexisting or terminal systemic disease). Finally, 38 patients were included in this study. The main characteristics and MRA findings of the patients are summarized in Table 1. Among 38 patients, the presence of FVH was observed in 31 patients (81.6%). There was no significant difference regarding sex and age between FVH-negative and FVH-positive groups (*p* > 0.05). The agreement for the detection of stenoses between the two raters was good (*κ* = 0.812). The major locations of stenosis were ICA and M1 segment of middle cerebral artery (MCA M1) in each group (for FVH-positive group, ICA: MCA M1 = 57.1% vs. 42.9%; for FVH-negative group, ICA: MCA M1 = 52.2% vs. 47.8%), and no significant difference was found (*p*=0.999). MRA showed that the degree of stenosis between the two groups was similar (*p*=0.826) (Figures 2 and 3).

Details of perfusion data of patients are presented in Table 2. The hypoperfusion regions, MTT, and CBF values were significantly different between the FVH-negative and FVH-positive groups, but there was no significant difference in CBV value. Compared with the FVH-negative group, the FVH-positive group, which presented with more severe impaired haemodynamics, showed a higher MTT (*p*=0.002) and lower CBF (*p*=0.021), as well as more extensive regions of hypoperfusion (*p*=0.002).

**Figure 4:**
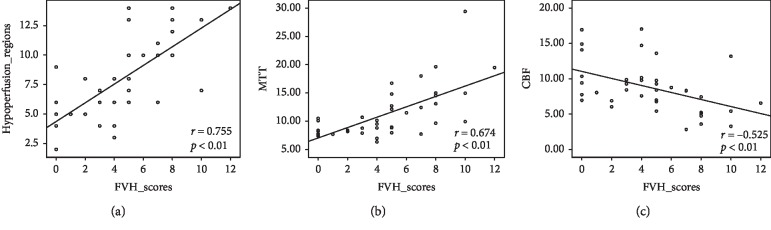
Scatter plots showed the hypoperfusion regions (a) and MTT (b) values were positively correlated with FVH scores (*r* = 0.755 and 0.674, respectively, *p* < 0.01); a moderate negative correlation was found between CBF (c) and FVH scores (*r* = −0.525, *p* < 0.01).

The Spearman rank correlation coefficients between the FVH score, stenosis grade, and perfusion parameters are shown in Table 2. Scatter plots showed that significant positive correlations were found between hypoperfusion regions, MTT, and FVHs scores (*r* = 0.755 and 0.674, respectively, *p* < 0.01); a moderate negative correlation was found between CBF and FVHs scores (*r* = −0.525, *p* < 0.01) (Figure 4), whereas the degree of artery stenosis revealed no significant correlation with FVH scores (*r* = 0.253, *p* > 0.05).

## 4. Discussion

According to previous studies, only 16.7–39.6% of TIA patients showed FVH when MRI was performed within 24 hours [5, 11, 12]. However, in our study, the presence of FVH was observed in 31 patients (81.6%). The selection of cases may explain this discrepancy. All of our subjects were hospitalized patients and most of them had severe stenoses or occlusions, as well as more severe symptoms than those of outpatients. However, previous studies enrolled an unselected series of consecutive patients who were suspected of TIAs. Because FVHs were proved as a transient sign and correlated with symptom resolution, the prior report may have underestimated the frequency of their occurrence [13].

In this study, we found that there was obvious arterial stenosis in all of TIA patients, regardless of whether they showed signs of FVH; meanwhile, no significant difference in the degree of stenosis was found between the FVH-negative and FVH-positive groups. That finding means that the severity of arterial stenosis did not predict the presence and the extent of FVH. In other words, arterial stenosis is a necessary, but not sufficient, condition for the presence of FVH.

Several studies have suggested that perfusion weighted imaging (PWI) lesions are most likely to be detected in TIA patients that have significant carotid stenosis or an intracranial occlusion that can be visualized on MRA [14–16]. Our perfusion findings concurred with previous studies. Perfusion imaging demonstrated varying degrees of hypoperfusion in all subjects. Moreover, compared with the FVH-negative group, the patients in the FVH-positive group showed more severe haemodynamic impairment, including not only perfusion parameters (CBF, CBV, and MTT) but also the involved topography.

Our results revealed that the FVH score was intensely associated with the areas of hypoperfusion and the severity of MTT prolongation, suggesting that the presence of FVH is representative of impaired cerebrovascular autoregulation. Based on these results, we consider that FVH can reasonably serve as a substitute for perfusion imaging when this is not available. Some similar conclusions have been drawn in studies concerning the FVH in acute infarct patients [17–20]. Toyoda et al. suggested that the presence of FVH could be used in place of PWI [17]. Liu et al. found that higher FVH-ASPECTS measured outside the DWI lesion is associated with good clinical outcomes in acute M1-middle cerebral artery occlusion patients with endovascular treatment [18]. In patients with distal FVHs, there were large DWI-PWI mismatches, indicating that these patients had large areas of salvageable brain parenchyma [19]. Therefore, we deduced that FVH could serve as good collateral evidence for haemodynamic impairment in patients with TIA.

There were several limitations for our study: (1) The sample size was small. (2) Because of limitations on CT modality, the size of perfusion coverage was not the whole brain; however, through optimization of the position of the region of interest, the depiction of hypoperfusion could be improved. (3) Clinical or functional outcome was not available for the study. Future studies should address the question of whether the presence of FHV is related to both tissue outcome and clinical outcome.

In conclusion, the presence of FVH showed no clear relationship with the severity of arterial stenosis. FVH could be interpreted as a marker of altered haemodynamics in patients with TIA of the carotid artery system. The results of this study suggest that the presence of FVH and its involved topography may help to increase the yield of MRI for the confirmation of haemodynamic impairment in patients with TIA of the carotid system.

## Figures and Tables

**Table 1:** Demographic and imaging characteristics of patients.

	FVH-positive (*n* = 31)	FVH-negative (*n* = 7)	*p* value
Gender			0.425
Male	19 (61.3%)	3 (42.9%)	
Female	12 (38.7%)	4 (57.1%)	
Age (years)	68 (64, 73)	64 (54, 65)	0.036^*∗*^
Degree of stenosis			0.826
Grade 1	2 (6.5%)	0 (0%)	
Grade 2	2 (6.5%)	1 (14.3%)	
Grade 3	18 (58.0%)	4 (57.1%)	
Grade 4	9 (29.0%)	2 (28.6%)	
Number of stenosis	1 (1, 2)	1 (1, 1)	0.394
Watershed regions	2 (2, 3)	2 (2, 2)	0.623
Hypoperfusion regions	8 (6, 13)	4 (4, 6)	0.005^*∗*^
MTT	10.137 (8.417, 14.820)	8.258 (7.512, 10.108)	0.037^*∗*^
CBF	8.052 (5.440, 9.756)	10.357 (7.758, 14.905)	0.030^*∗*^
CBV	1.005 (0.701, 1.210)	1.134 (0.971, 1.296)	0.337

CBF, cerebral blood flow; CBV, cerebral blood volume; DWI, diffusion weighted imaging; FHV, fluid-attenuated inversion recovery vascular hyperintensity; MTT, mean transit time.

**Table 2:** Spearman rank correlations.

		Hypoperfusion regions	MTT	CBF	Stenosis grade
FVH scores	Spearman correlation	0.755^*∗∗*^	0.674^*∗∗*^	−0.525^*∗∗*^	0.253
	Sig. (2-tailed)	0.000	0.000	0.001	0.125

FHV, fluid-attenuated inversion recovery vascular hyperintensity; CBF = cerebral blood flow; CBV = cerebral blood volume; MTT = mean transit time. ^*∗∗*^Correlation is significant at the 0.01 level (2-tailed).

## Data Availability

The data used to support the findings of this study are available from the corresponding author upon request.

## Abbreviations

ASPECTS: Alberta Stroke Programme Early Computerized Tomography Score
CBF: Cerebral blood flow
CBV: Cerebral blood volume
CT: Computed tomography
DWI: Diffusion weighted imaging
FLAIR: Fluid-attenuated inversion recovery
FVH: Fluid-attenuated inversion recovery vascular hyperintensity
Gd-DTPA: Gadolinium-diethylenetriamine penta-acetic acid
ICA: Internal carotid artery
MCA: Middle cerebral artery
MRA: Magnetic resonance angiography
MRI: Magnetic resonance imaging
MTT: Mean transit time
NASCET: North American Symptomatic Carotid Endarterectomy Trial
PWI: Perfusion weighted imaging
ROI: Region of interest
SE-EPI: Spin echo-echo planar imaging
TIA: Transient ischemic attack.
